# Circulating Bacterial-Derived DNA Fragment Level Is a Strong Predictor of Cardiovascular Disease in Peritoneal Dialysis Patients

**DOI:** 10.1371/journal.pone.0125162

**Published:** 2015-05-26

**Authors:** Cheuk-Chun Szeto, Bonnie Ching-Ha Kwan, Kai-Ming Chow, Jeffrey Sung-Shing Kwok, Ka-Bik Lai, Phyllis Mei-Shan Cheng, Wing-Fai Pang, Jack Kit-Chung Ng, Michael Ho-Ming Chan, Lydia Choi-Wan Lit, Chi-Bon Leung, Philip Kam-Tao Li

**Affiliations:** 1 Carol & Richard Yu Peritoneal Dialysis Research Centre, Department of Medicine & Therapeutics, Prince of Wales Hospital, The Chinese University of Hong Kong, Shatin, Hong Kong, China; 2 Department of Chemical Pathology, Prince of Wales Hospital, The Chinese University of Hong Kong, Shatin, Hong Kong, China; Cardiff University School of Medicine, UNITED KINGDOM

## Abstract

**Background:**

Circulating bacterial DNA fragment is related to systemic inflammatory state in peritoneal dialysis (PD) patients. We hypothesize that plasma bacterial DNA level predicts cardiovascular events in new PD patients.

**Methods:**

We measured plasma bacterial DNA level in 191 new PD patients, who were then followed for at least a year for the development of cardiovascular event, hospitalization, and patient survival.

**Results:**

The average age was 59.3 ± 11.8 years; plasma bacterial DNA level 34.9 ± 1.5 cycles; average follow up 23.2 ± 9.7 months. At 24 months, the event-free survival was 86.1%, 69.8%, 55.4% and 30.8% for plasma bacterial DNA level quartiles I, II, III and IV, respectively (p < 0.0001). After adjusting for confounders, plasma bacterial DNA level, baseline residual renal function and malnutrition-inflammation score were independent predictors of composite cardiovascular end-point; each doubling in plasma bacterial DNA level confers a 26.9% (95% confidence interval, 13.0 – 42.5%) excess in risk. Plasma bacterial DNA also correlated with the number of hospital admission (r = -0.379, p < 0.0001) and duration of hospitalization for cardiovascular reasons (r = -0.386, p < 0.0001). Plasma bacterial DNA level did not correlate with baseline arterial pulse wave velocity (PWV), but with the change in carotid-radial PWV in one year (r = -0.238, p = 0.005).

**Conclusions:**

Circulating bacterial DNA fragment level is a strong predictor of cardiovascular event, need of hospitalization, as well as the progressive change in arterial stiffness in new PD patients.

## Introduction

Patients with chronic kidney disease (CKD) or on long-term dialysis are at high risk of developing cardiovascular disease (CVD) [1–3]. Longitudinal studies have established that CVD events occur more frequently than renal events in CKD, and CVD mortality rates are in fact higher than the rates of reaching end-stage renal disease (ESRD) [4,5]. Although CVD shares many similar risk factors with CKD, such as diabetes and hypertension [6], CKD remains an independent risk factor for CVD after accounting for traditional risk factors [7].

It is now recognized that systemic inflammation plays a key role in atherosclerosis [8] and is an important contributor to CVD morbidity and mortality in CKD patients [9]. Nearly 50% of CKD or dialysis patients have evidence of systemic inflammation [10–12]. Previous studies showed that circulating lipopolysaccharide (LPS) constitutes a strong risk factor of early atherogenesis in patients with chronic bacterial infections [13]. Epidemiological studies show that even a low level endotoxemia constitutes a strong risk factor for the development of atherosclerosis [14]. There is now evidence that elevated circulating LPS level is common in CKD patients. The intestinal mucosa barrier is impaired and bacterial translocation occurs in experimental uremia [15]. Translocation of bowel flora is a cause of gram negative peritonitis in peritoneal dialysis (PD) patients [16]. A number of previous studies showed that LPS is detectable in the serum of many dialysis patients, and serum LPS level correlates with the severity of systemic inflammation and features of atherosclerosis [17–19].

In addition to LPS, which is a bacterial cell wall component, other bacterial fragments could also be detected in human circulation, and bacterial-derived DNA fragment is the most easily and consistently detectable component. Because most bacteria contain the highly conserved 16S rRNA gene in the genome, the sequence could be easily detected and discerned from human DNA. A previous study showed that circulating bacterial-derived DNA fragments were present in around 20% of hemodialysis patients, and circulating bacterial-derived DNA fragments are associated with higher levels of C-reactive protein and IL-6 in these patients [20]. Our previous study showed that circulating bacterial DNA level correlated with the degree of systemic inflammatory state in PD patients [21]. In the present study, we determine the relation between circulating bacterial-derived DNA fragment and cardiovascular disease in PD patients.

## Patients and Methods

### Patient selection

The study is approved by Joint Chinese University of Hong Kong—New Territories East Cluster Clinical Research Ethics Committee (approval number CRE-2010.375). All patients provided signed written informed consent before participation in this study. We recruited 191 consecutive new PD patients, with age 18 to 80 years, from July 2010 to December 2012. Patients who were unlikely to survive for 6 months, who were planned to have elective living donor transplant or transfer to other renal center within 6 months were excluded. After written informed consent, a panel of comorbid conditions was recorded. The modified Charlson’s comorbidity index, which was validated in PD patients [17], was used to calculate a comorbidity score. In addition to the routine blood tests for electrolyte, fasting glucose and lipid levels, serum levels of bacterial DNA fragment, LPS, C-reactive protein (CRP), and procalcitonin as markers of systemic inflammation were measured when the patient was stable without clinical evidence of peritonitis or systemic infection. Baseline clinical data were recorded by chart review. These included age, sex, underlying renal disease, and PD regimen. Serum C-reactive protein (CRP) was measured by the Tina-quant CRP (Latex) ultra-sensitive assay (Roche Diagnostics GmbH, Mannheim, Germany). Serum procalcitonin level was determined by the Elecsys BRAHMS PCT assay (Roche Diagnostics, Indianapolis, IN, USA) according to the manufacturer’s instruction. Nutritional assessment, 24-hour dialysate and urine collection for assessment of dialysis adequacy and residual renal function, arterial pulse wave velocity study, and bioimpedance spectroscopy were performed at recruitment and then 12 months later. In addition, baseline standard peritoneal equilibration test (PET) was also performed.

### Plasma bacterial DNA fragment

The method of bacterial DNA amplification has been described previously [19,20]. Briefly, DNA from 200 μl aliquot of EDTA-treated whole blood was extracted using the EZ1 DNA tissue kit and BioRobot EZ1 with the EZ1 bacteria card (Qiagen), according to the manufacturer’s instructions. Purified DNA was eluted in 50 μl of elution buffer before amplification. Universal primers used for polymerase chain reaction (PCR) amplification of the bacterial 16S rRNA gene were p16SrRNA+ and p16SrRNA-, which are able to amplify DNA from either Gram positive or Gram negative bacteria. Aliquots of 20-μl DNA samples were used for amplification in a 50-μl PCR reaction mixture. All samples were run in triplicates. Since plasma was directly used as the template and there is no intrinsic housekeeping gene for comparison, the number of PCR cycles at which bacterial DNA could be detected is reported.

### Plasma LPS level

The method of plasma LPS quantification has been described previously [17]. Briefly, plasma samples were diluted to 20% with endotoxin-free water and then heated to 70°C for 10 min to inactivate plasma proteins. We then quantified plasma LPS with a commercially available Limulus Amebocyte Lysate assay (Cambrex, Verviers, Belgium) according to the manufacturer's protocol. The detection limit of this assay was 0.01 EU/ml. Samples with LPS level below the detection limit were taken as 0 EU/ml. All samples were run in duplicate and background subtracted.

### Study of peritoneal transport

Standard peritoneal permeability test (PET) was performed by the method of Twardowski and has been described previously [22]. Briefly, a 4-hour dwell study was carried out with 2 liters of dextrose 2.5% dialysis fluid (Dianeal, Baxter-Travenol, Deerfield, IL). Dialysate creatinine and glucose levels at 0, 2 and 4 hours, plasma creatinine and glucose levels at 2 hour are measured. Drainage and ultrafiltration volumes (UF) at 4 hour are documented. Dialysate-to-plasma ratios of creatinine (D/P) at 0, 2, and 4 hours are calculated after correction of glucose interference. Mass transfer area coefficients of creatinine (MTAC) normalized for body surface area (BSA) is calculated by the formula described by Krediet [23]. Body surface area (BSA) is determined from body weight and height by nomogram [24].

### Dialysis adequacy and nutritional indices

The method of dialysis adequacy assessment has been described previously [25]. Briefly, 24-hour urine and dialysate collection was performed to calculate total Kt/V. Nutritional status was represented by serum albumin level, subjective global assessment (SGA) comprehensive malnutrition-inflammation score (MIS), normalized protein nitrogen appearance (NPNA), and fat-free edema-free body mass (FEBM). For SGA, the 4-item 7-point scoring system, which was validated in PD patients [26], was used. The calculation of MIS was described previously [27]. Briefly, MIS consists of 4 main parts and 10 components, all scored from 0 (normal) to 3 (very severe). The total score ranged from 0 to 30. NPNA was calculated by the modified Bergstrom’s formula [28]. FEBM was determined by creatinine kinetics according to the formula of Forbes and Bruining, a method that is recommended by the standard dialysis practice guideline [29].

### Pulse wave velocity study

Pulse wave velocity (PWV), an index of arterial stiffness, is measured using an automatic computerized recorder and the results are analyzed using the Complior® SP program (Artech Medical, France). The method of PWV measurement has been described previously [30]. Briefly, pressure-sensitive transducers are placed over the neck (carotid artery), wrist (radial artery) and groin (femoral artery) with the patient in the supine position. PWV of the carotid-femoral and carotid-radial territory is calculated by dividing the distance between the sensors by the time corresponding to the period separating the start of the rising phase of the carotid pulse wave and that of the femoral and also the radial pulse waves.

### Clinical follow up and outcome measures

After recruitment, patients will be followed every 8 weeks, or more frequently if clinically indicated, for at least one year. The clinical management will not be affected by the study. The pre-defined primary end point of this study is a composite one that consists of cardiovascular death, non-fatal myocardial infarction or stroke, hospital admission for unstable angina, coronary intervention, congestive heart failure, transient ischemic attack, cerebrovascular accident, or peripheral vascular disease that require surgical reconstruction or amputation. Event-free survival is then reported, with the pre-defined primary end point as event and non-cardiovascular deaths, transfer to hemodialysis, and transplantation as censoring observations. After the study has completed, we further defined a composite cardiovascular end point that encompasses all of the above but excludes congestive heart failure. Secondary end points include number of hospital admission and duration of hospitalization during the study period, cardiovascular mortality, all-cause mortality, technique survival, and peritonitis-free survival. Technique failure is defined as transfer to long-term hemodialysis. Survival data were censored on 31 December 2013.

### Statistical analysis

Statistical analysis was performed by SPSS for Windows software version 15.0 (SPSS Inc., Chicago, IL). Data are expressed as means ± SD unless otherwise specified. Data were compared by Student’s t test, Chi square test or Pearson’s correlation coefficient as appropriate. The relationship between plasma bacterial DNA and LPS levels and the primary composite end point or survival was analyzed by stratifying patients into quartiles according to the bacterial DNA or LPS level. Survival rates were analyzed using Kaplan—Meier survival curves. The Cox proportional hazards model was used to adjust for potential confounders and identify independent predictors of the composite cardiovascular end-point, patient survival, and technique survival. In addition to baseline plasma bacterial DNA level, the Cox models were constructed by age, Charlson’s comorbidity score, carotid-femoral PWV, serum CRP, serum albumin, total Kt/V, NPNA and residual GFR. These parameters were selected for the construction of the Cox model because of their importance in determining the survival of PD patients. The assumption of proportional hazard was tested and confirmed by graphical methods. All variables were added independently into the Cox model. Backward stepwise elimination was applied to remove insignificant variables. Interaction between variables was excluded as correlation matrix shows only modest internal correlations and additional direct testing in the final model.

The number of hospital admission and duration of hospitalization are compared between plasma bacterial DNA level quartiles after adjusted for the duration of follow up because the data were significantly skewed. Since plasma bacterial DNA level is a continuous variable, the log-linear model was then used to analyze hospitalization [31,32]. The clinical variables used for analysis were similar to those for survival analysis. A value of p < 0.05 was considered statistically significant. All probabilities were two-tailed.

## Results

We studied 191 consecutive new PD patients. The demographic, baseline clinical and biochemical information are summarized in Tables 1 and 2, respectively. The average level of plasma bacterial DNA was 34.9 ± 1.5 cycles; plasma LPS level was 0.72 ± 0.34 EU/ml.

**Table 1:** Baseline demographic and clinical data.

Plasma bacterial DNA quartile	I	II	III	IV	P value
No. of patients	48	48	48	47	
Sex (M:F)	23:25	26:22	28:20	34:13	p = 0.13
Age (years)	60.9 ± 10.1	60.0 ± 10.8	57.9 ± 13.8	58.3 ± 12.3	p = 0.6
Body height (cm)	160.6 ± 8.8	162.3 ± 9.0	161.9 ± 8.9	161.2 ± 8.5	p = 0.9
Body weight (kg)	66.9 ± 16.7	63.7 ± 15.1	63.6 ± 13.6	68.1 ± 15.7	p = 0.4
Blood pressure (mmHg)					
Systolic	142.0 ± 18.7	144.8 ± 24.6	134.8 ± 20.6	142.1 ± 17.8	p = 0.12
Diastolic	76.7 ± 11.0	76.7 ± 13.7	73.6 ± 11.9	75.1 ± 10.8	p = 0.5
Renal diagnosis, no. of cases (%)					p = 0.07
Glomerulonephritis	10 (20.8%)	11 (22.9%)	15 (31.3%)	10 (21.3%)	
Diabetic nephropathy	25 (52.1%)	22 (45.8%)	17 (35.4%)	27 (57.4%)	
Polycystic kidney	4 (8.3%)	0	1 (2.1%)	5 (10.6%)	
Hypertensive nephrosclerosis	3 (6.3%)	7 (14.6%)	6 (12.5%)	2 (4.3%)	
Obstructive uropathy	4 (8.3%)	2 (4.2%)	1 (2.1%)	1 (2.1%)	
Others / unknown	2 (4.2%)	6 (12.5%)	8 (16.7%)	2 (4.3%)	
Pre-existing vascular disease, no. of cases (%)					
Diabetes	28 (58.3%)	24 (50.0%)	26 (54.2%)	30 (63.8%)	p = 0.5
Coronary heart disease	11 (22.9%)	3 (6.3%)	10 (20.8%)	8 (17.0%)	p = 0.9
Cerebrovascular disease	11 (22.9%)	10 (20.8%)	8 (16.7%)	9 (19.1%)	p = 0.5
Peripheral vascular disease	3 (6.3%)	1 (2.1%)	2 (4.2%)	1 (2.1%)	p = 0.4
Charlson’s comorbidity index	5.63 ± 2.19	5.06 ± 1.91	4.73 ± 2.16	5.44 ± 2.30	p = 0.4
Plasma bacterial DNA level (PCR cycles)	36.3 ± 0.4	35.6 ± 0.2	34.8 ± 0.3	32.8 ± 1.6	p < 0.0001

PD, peritoneal dialysis; PCR, polymerase chain reaction. Patients were divided to quartiles of plasma bacterial DNA. Quartile I had the lowest while quartile IV the highest plasma bacterial DNA level. Note that a higher PCR cycle number indicates a lower level of bacterial DNA. Data are compared by Chi square test or one way analysis of variance (ANOVA) as appropriate.

**Table 2:** Baseline biochemical data and dialysis prescription.

Plasma bacterial DNA quartile	I	II	III	IV	P value
No. of patients	48	48	48	47	
Malnutrition inflammation score	6.4 ± 4.5	6.1 ± 3.2	7.3 ± 3.8	6.4 ± 3.9	p = 0.7
Subjective Global Assessment	5.3 ± 0.9	5.6 ± 0.7	5.3 ± 0.9	5.4 ± 1.0	p = 0.5
Hemoglobin (g/dL)	9.7 ± 2.0	9.2 ± 1.0	9.4 ± 1.7	9.3 ± 1.4	p = 0.6
Serum albumin (g/L)	34.4 ± 5.3	34.3 ± 3.7	33.9 ± 4.4	35.0 ± 5.5	p = 0.8
Lipid profile					
Total cholesterol (mmol/l)	4.77 ± 1.21	4.88 ± 1.37	4.97 ± 1.19	4.69 ± 1.66	p = 0.8
Triglyceride (mmol/l)	1.56 ± 0.87	1.63 ± 0.97	1.78 ± 0.95	1.74 ± 1.14	p = 0.7
LDL cholesterol (mmol/l)	2.68 ± 1.04	2.89 ± 1.11	3.00 ± 1.07	2.71 ± 1.24	p = 0.5
HDL cholesterol (mmol/l)	1.35 ± 0.44	1.28 ± 0.36	1.24 ± 0.38	1.21 ± 0.38	p = 0.4
Peritoneal transport					
Ultrafiltration volume (L)	0.35 ± 0.19	0.33 ± 0.23	0.37 ± 0.22	0.29 ± 0.18	p = 0.3
D/P creatinine at 4 hour	0.66 ± 0.13	0.69 ± 0.16	0.66 ± 0.13	0.63 ± 0.16	p = 0.2
MTAC creatinine (ml/min/1.73m2)	9.9 ± 4.6	11.8 ± 6.8	10.3 ± 5.0	9.4 ± 5.4	p = 0.2
Dialysis adequacy					
Weekly total Kt/V	2.12 ± 0.47	2.03 ± 0.57	2.15 ± 0.63	2.26 ± 0.63	p = 0.3
Residual GFR (ml/min/1.73m2)	3.82 ± 2.45	3.17 ± 2.65	3.50 ± 3.01	4.64 ± 2.59	p = 0.07
NPNA (g/kg/day)	1.20 ± 0.25	1.09 ± 0.22	1.13 ± 0.22	1.14 ± 0.26	p = 0.14
FEBM (%)	39.5 ± 10.9	44.2 ± 14.3	43.8 ± 15.0	39.0 ± 11.1	p = 0.11
Machine-assisted PD, no. of cases (%)	9 (18.8%)	6 (12.5%)	5 (10.4%)	8 (17.0%)	p = 0.6
Use icodextrin, no. of case (%)	12 (25.0%)	15 (31.3%)	15 (31.3%)	15 (31.9%)	p = 0.8
Daily exchange volume (L/day)					
0 month	6.2 ± 0.6	6.1 ± 0.5	6.2 ± 0.6	6.3 ± 0.7	p = 0.7
12 month	6.4 ± 0.9	6.4 ± 0.8	6.5 ± 1.3	6.5 ± 1.1	p = 0.9
Glucose load (g/day)					
0 month	97.7 ± 29.9	95.8 ± 26.6	99.7 ± 34.0	98.1 ± 33.7	p = 0.9
12 month	108.0 ± 33.6	112.2 ± 42.8	115.2 ± 44.4	114.4 ± 46.0	p = 0.9

HDL, high density lipoprotein; LDL, low density lipoprotein; D/P, dialysate-to-plasma concentration ratio of creatinine; MTAC, mass transfer area coefficient; GFR, glomerular filtration rate; NPNA, normalized protein nitrogen appearance; FEBM, fat-free edema-free body mass by creatinine kinetics. Patients were divided to quartiles of plasma bacterial DNA. Quartile I had the lowest while quartile IV the highest plasma bacterial DNA level. Data are compared by one way analysis of variance (ANOVA).

### Relation with systemic inflammatory markers

There was a modest but statistically significant correlation between plasma bacterial DNA level and plasma LPS level (r = -0.410, p < 0.0001). Both plasma bacterial DNA and plasma LPS levels significantly correlated with serum CRP level (r = -0.299 and r = 0.313 respectively, p < 0.0001 for both). On the other hand, neither plasma bacterial DNA nor LPS levels correlated with serum procalcitonin level (details not shown). Plasma bacterial DNA level had modest but significant correlations with the malnutrition-inflammation score (r = -0.179, p = 0.016) as well as SGA score (r = 0.165, p = 0.026). Neither plasma bacterial DNA or plasma LPS level correlated with the Charlson’s comorbidity score, peritoneal transport status, or dialysis adequacy indices (details not shown).

### Relation with composite cardiovascular end point

**Fig 1:**
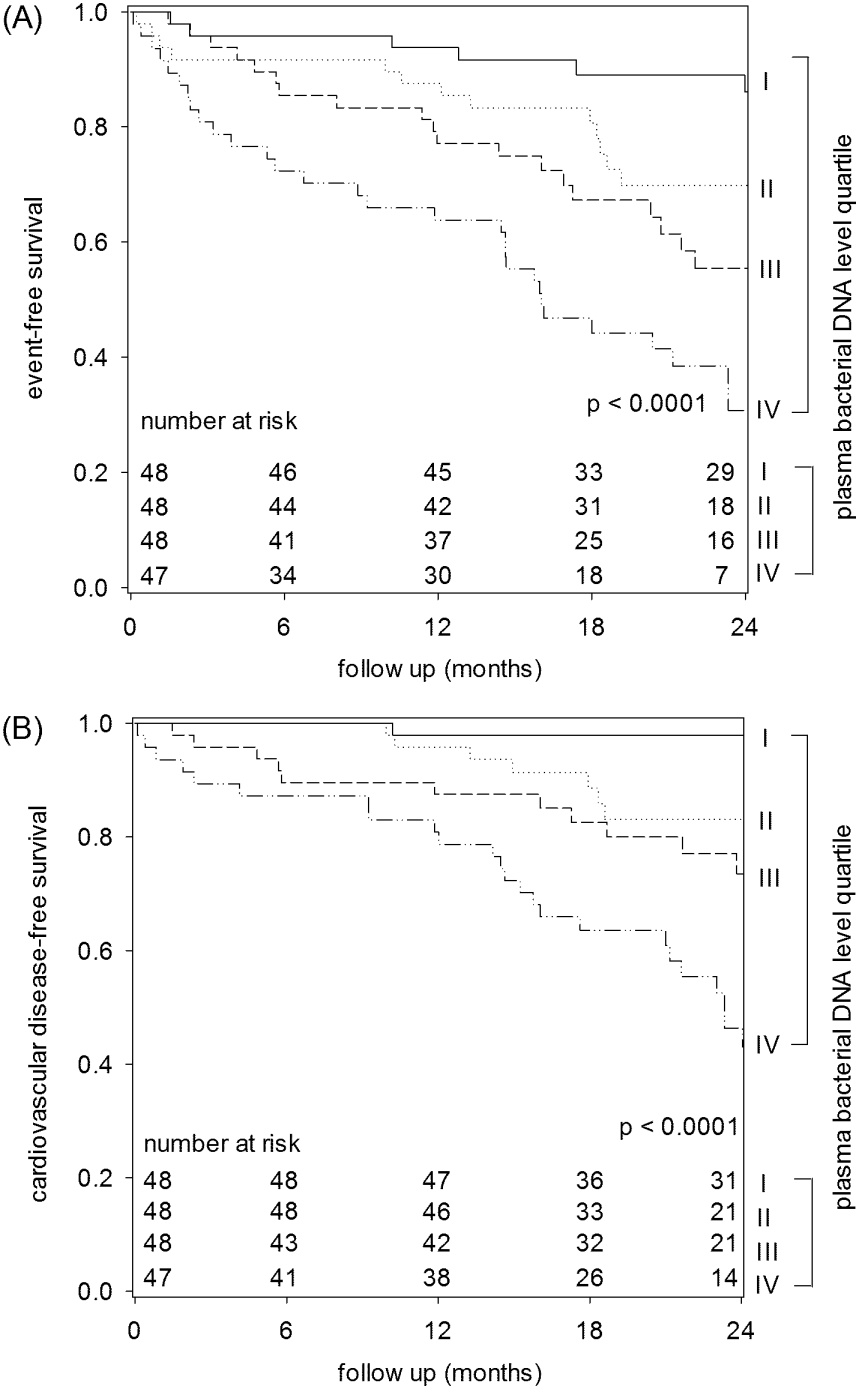
Kaplan-Meier plot of (A) event-free survival; and (B) cardiovascular disease-free survival (excluding congestive heart failure). Patients were divided to quartiles of plasma bacterial DNA. Quartile I had the lowest while quartile IV the highest plasma bacterial DNA level. Data are compared by the log rank test.

The average follow up was 23.2 ± 9.7 months. During this period, 80 patients (41.9%) developed cardiovascular events as defined by the primary composite end point. These include hospital admission for heart failure (48 cases), non-fatal stroke (14 cases), non-fatal myocardial infarction or acute coronary syndrome (11 cases), elective admission for coronary interventions (4 cases), and limb amputation for peripheral vascular disease (3 cases). At 24 months, the event-free survival was 86.1%, 69.8%, 55.4% and 30.8% for plasma bacterial DNA level quartiles I, II, III and IV, respectively (log rank test, p < 0.0001) (Fig 1A). After excluding admission for congestive heart failure, 62 patients (32.5%) developed the composite cardiovascular end point. The 24-month cardiovascular disease-free survival was 97.9%, 83.1%, 73.5% and 46.4% for plasma bacterial DNA level quartiles I, II, III and IV, respectively (log rank test, p < 0.0001) (Fig 1B). On the other hand, the event free survival at 24 months was 71.2%, 58.3%, 54.4% and 58.6% for plasma LPS level quartiles I, II, III and IV, respectively (p = 0.051). By multivariable analysis with the Cox proportional hazard model to adjust for confounders, plasma bacterial DNA level, baseline residual GFR and malnutrition-inflammation score were the independent predictors of the composite cardiovascular end-point (Table 3). In this model, each doubling in plasma bacterial DNA level confers a 26.9% (95% confidence interval, 13.0–42.5%) excess in risk of developing the composite cardiovascular end point.

**Table 3:** Cox proportional hazards model of composite cardiovascular end-point.

variable	AHR	95% CI	P value
plasma bacterial DNA	1.269	1.130–1.425	p < 0.0001
residual GFR	0.887	0.788–0.999	p = 0.047
MIS	1.097	1.001–1.204	p = 0.049

AHR, adjusted hazard ratio; CI, confidence interval; MIS, malnutrition inflammation score; GFR, glomerular filtration rate.

### Relation with survival and peritonitis

During the study period, 31 patients (16.2%) died. The causes of death were cardiac arrest (6 cases), coronary artery disease (3 cases), stroke (7 cases), peritonitis (4 cases), non-peritonitis infection (8 cases), cancer (2 cases), and liver failure (1 case). During this period, another 11 patients had kidney transplant, 7 were changed to long term hemodialysis, 1 transferred to other centers, and 1 had recovery of renal function. At 24 months, the overall patient survival was 90.9%, 76.2%, 84.7% and 87.6% for plasma bacterial DNA level quartiles I, II, III and IV, respectively (p = 0.2), while technique survival was 76.7%, 74.2%, 70.7% and 81.9%, respective (p = 0.3). There was no relation between plasma bacterial DNA level and peritonitis rate or peritonitis-free survival (details not shown). There was also no significant relation between plasma LPS level and patient or technique survival (details not shown).

### Relation with hospitalization

**Fig 2:**
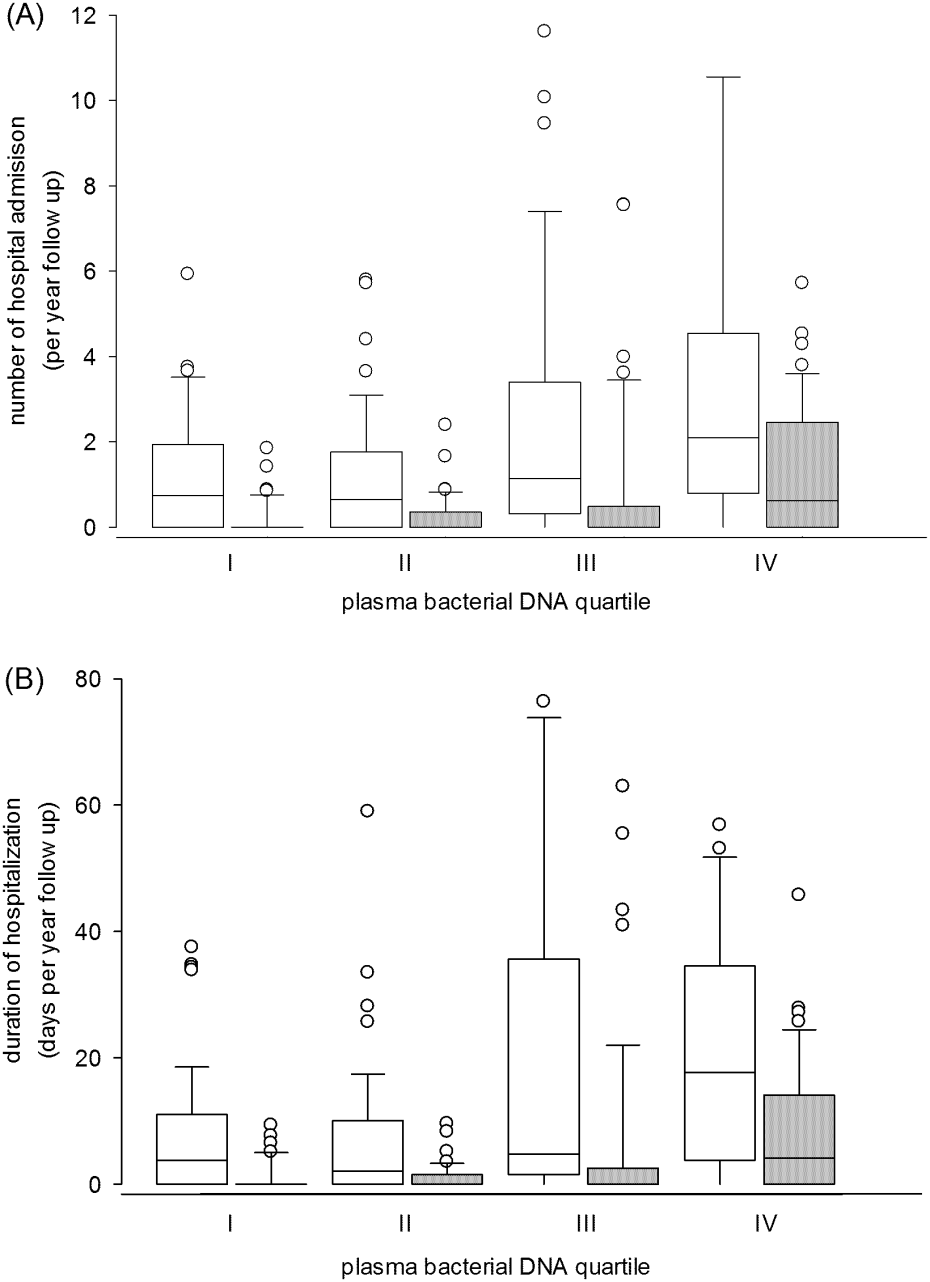
Comparison of (A) number of hospital admission; and (B) duration of hospitalization between quartiles of plasma bacterial DNA level. Quartile I had the lowest while quartile IV the highest plasma bacterial DNA level. (p < 0.0001 by Kruskal Wallis test for all comparisons between quartiles) (White box, hospitalization for all cause; hatched box, hospitalization for cardiovascular reasons.)

During the study period, there were altogether 600 hospital admissions, of which 185 admissions were for cardiovascular reasons; 51 patients (26.7%) did not require any hospital admission. The total duration of hospitalization was 4201 days, with 1327 days for cardiovascular reasons. The number of hospital admission and duration of hospitalization for cardiovascular reasons are compared between plasma bacterial DNA level quartiles and summarized in Fig 2. In short, plasma bacterial DNA significantly correlated with the number of hospital admission for cardiovascular reasons (r = -0.379, p < 0.0001) and duration of hospitalization for cardiovascular reasons (r = -0.386, p < 0.0001). The correlations between plasma bacterial DNA level and total number of hospital admission (r = -0.316, p < 0.0001) as well as total duration of hospitalization (r = -0.339, p < 0.0001) were also significant but less strong. Plasma LPS level also correlated with the number of hospital admission for cardiovascular reasons (r = 0.182, p = 0.012) and duration of hospitalization for cardiovascular reasons (r = -0.179, p = 0.013), but the degree of correlation was substantially lower than those of bacterial DNA level. By multivariable analysis with the log-linear regression model to adjust for confounders, plasma bacterial DNA level and malnutrition inflammation score were the only two independent predictors of hospitalization for cardiovascular reasons (Table 4).

**Table 4:** Independent predictors of hospitalization for cardiovascular reasons by log-linear model.

variable	e^COEF^	95%CI	P value
number of hospital admission			
plasma bacterial DNA level (2-fold)	1.222	1.133–1.318	p < 0.0001
MIS (1 point)	1.036	1.005–1.068	p = 0.022
duration of hospitalization			
plasma bacterial DNA level (2-fold)	1.323	1.157–1.512	p < 0.0001
MIS (1 point)	1.083	1.006–1.065	p = 0.033

CI, confidence interval; MIS, malnutrition inflammation score.

NB. e^COEF^ was the exponential coefficient indicating the relative number of hospital admission (per year) or duration of hospitalization (days per year of follow up) compared to the 2-fold lower of plasma bacterial DNA level (i.e. one extra threshold cycle of polymerase chain reaction), and 1 point less for MIS.

### Relation with arterial pulse wave velocity

**Fig 3:**
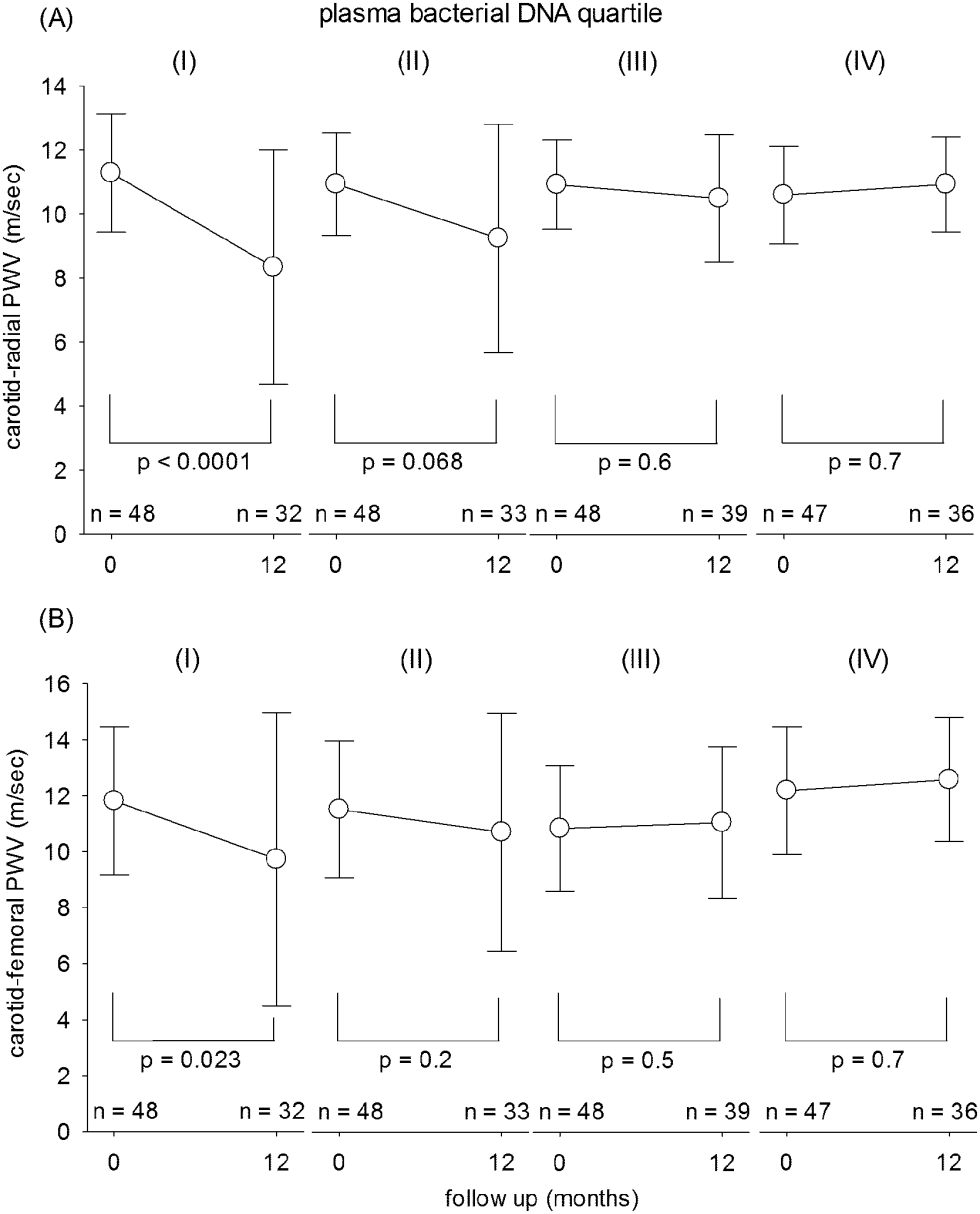
Comparison of (A) carotid-radial; and (B) carotid-femoral pulse wave velocity (PWV) between quartiles of plasma bacterial DNA level. Quartile I had the lowest while quartile IV the highest plasma bacterial DNA level. P values depicted are computed by paired Student’s t test.

For the entire cohort, carotid-radial PWV decreased during the first 12 months of PD from 10.9 ± 1.6 to 9.7 ± 3.0 m/sec (p < 0.0001), while carotid-femoral PWV decreased from 11.9 ± 2.5 to 11.3 ± 4.1 m/sec (p = 0.049). The change in arterial pulse wave velocity over 12 months are compared between plasma bacterial DNA level quartiles and summarized in Fig 3. In short, baseline plasma bacterial DNA level did not correlate with baseline PWV. However, plasma bacterial DNA level significantly correlated with the change in carotid-radial (r = -0.238, p = 0.005), but not carotid-femoral (r = -0.107, p = 0.2) PWV, during the first 12 months of PD, and it had a modest but significant inverse correlation with carotid-radial (r = -0.207, p = 0.014) and carotid-femoral (r = -0.238, p = 0.005) at PWV at 12 months. There is no significant difference in baseline carotid-radial or carotid-femoral PWV between patients who could and could not complete 12 months of follow up (details not shown). Similarly, plasma LPS level also correlated with the change in carotid-radial (r = 0.194, p = 0.022), but not carotid-femoral (r = 0.113, p = 0.2) PWV, during the first 12 months of PD.

## Discussion

In the present study, we found that circulating bacterial DNA fragment level has a modest correlation with markers of systemic inflammation. More importantly, circulating bacterial DNA fragment level is a strong predictor of cardiovascular event, need of hospitalization, as well as the progressive change in arterial stiffness (as reflected by arterial pulse wave velocity) in new PD patients.

The result of our present study is in line with previous reports. For example, Bossola et al [20] showed that circulating bacterial DNA fragments is a marker of systemic inflammation in chronic hemodialysis patients. In our previous study, we found that plasma bacterial DNA level correlated with the degree of systemic inflammatory state in PD patients, but there was no association between plasma bacterial DNA level and patient survival or peritonitis-free survival after adjusting for confounding factors [21]. The magnitude of correlation between plasma bacterial DNA level and plasma LPS or CRP level is similar between the two studies. In this study, we chose CRP as the marker of systemic inflammation because its relation with cardiovascular disease in dialysis patient is well reported [33]. Unfortunately, we did not measure the plasma level of other cytokines (e.g. interleukin-6), which has been shown to add significantly greater predictive power for all-cause and cardiovascular death in dialysis patients [34]. Contrary to the correlation with CRP level, we did not find any significant correlation between plasma bacterial DNA and procalcitonin levels. Current evidence suggests that serum procalcitonin is a specific marker of active bacterial infection, while CRP level denotes systemic inflammatory state [35]. Our results further support the notion that the presence of circulating bacterial fragment in PD patient is not the result of occult bacterial infection. Similar to the previous study [21], our present one did not found any correlation between plasma bacterial DNA level and patient or technique survival. Nonetheless, the present study supports the hypothesis that circulating bacterial DNA fragment contributes to the pathogenesis of cardiovascular disease in PD patients, which is an area not explored in the previous study [21].

Several previous studies showed that plasma LPS (i.e. bacterial cell wall fragment) level is related to systemic inflammation, erythropoietin resistance, and cardiovascular disease in chronic kidney disease [17,18,36]. In patients receiving hemodialysis, systemic circulatory stress induced by the dialysis procedure, as well as recurrent regional ischemia, may lead to endotoxin translocation from the gut, resulting in a systemic inflammatory state, progressive malnutrition, cardiac injury, and reduced survival [18]. However, the prognostic value of plasma LPS level in PD patients is less certain. We have previously observed a better technique survival and an insignificant trend of fewer cardiovascular events in new PD patients with a higher baseline plasma LPS level [37]. In our present study, we also observed a modest but significant relation between plasma LPS level and systemic inflammation as well as subsequent hospitalization, but the magnitude of correlation was substantially less than that with plasma bacterial DNA level.

The mechanism of bacterial fragment induced inflammatory state and atherosclerosis is incompletely understood. Current evidence suggests that metabolic alterations of uremia favor pathogen overgrowth in the gut and alteration in bowel permeability, resulting in an increased translocation of bacterial components [38]. This process then activates innate immunity and systemic inflammation. Our study does not show that bacterial DNA fragment is the only source of microbial inflammatory trigger. We believe all types of circulating bacterial fragments contribute to the pathogenesis of cardiovascular disease in PD patients, and, as compare to LPS level, plasma bacterial DNA level seems to be a superior marker of circulating load of bacterial fragment. Theoretically, plasma bacterial DNA level may represent a more accurate measurement of the load of circulating bacterial fragment than LPS level because LPS is by and large the cell wall component of Gram negative bacteria, while the bacterial DNA assay we used detects both Gram positive and Gram negative species.

Based on our result, it is tempting to hypothesize therapeutic measures that lower circulating bacterial DNA (as well as other microbial fragments) levels may be an effective means for cardiovascular protection. Interventions that manipulate the gut microbiota, such as pre- or probiotics, have been proposed to correct the immune dysregulation in renal failure and to prevent complications related to uremia [38,39]. Alternatively, interventions that aim to neutralize bacterial endotoxins or adsorb gut-derived uremic toxins have been considered [39]. Recently, John et al [19] reported that in elderly patients, improvement of their cardiovascular status with optimized antihypertensive therapy is associated with a significant reduction in the circulating LPS level. We have previously showed that using ultrapure dialysate for hemodialysis effectively reduces circulating LPS but not bacterial DNA level in hemodialysis patients [40]. However, none of these measures has been proved to improve hard clinical end points (for example, reducing cardiovascular event or hospitalization). Further clinical trials in this area are much needed.

There are a number of inadequacies of our present study. First, the sample size estimation was based on the primary composite end point and is therefore inadequate to determine the effect of bacterial DNA level on patient survival, and the negative result of survival analysis in our study may represent type 2 statistical error. The sample size is too small to test the effect of each individual factor. Nonetheless, a post hoc pooled analysis that also include patients in our previous study [21], with a total of 491 patients, does not reveal any trend of survival difference between patients with different plasma bacterial DNA level quartiles (S1 Fig). As a single center study, one also needs to be cautious about the generalizability of our result. Further large cohort studies are needed to validate the result, especially in patients from other ethnic groups.

In the present study, we did not determine the serial change of plasma bacterial DNA level with time, although our previous study showed that plasma bacteria DNA levels remains static over 12 months in PD patients [21]. In addition, we do not have detailed information on the fluid status of our patients. Since systemic fluid overload could either be the cause or effect of bacterial fragment translocation from the gut, it would be interesting to explore the relation between plasma bacterial DNA level and body fluid status (for example, by bioimpedance spectroscopy). Although a substantial proportion of the events that were counted as the primary composite end point were hospitalization for heart failure, the result remains similar after excluding fluid overload as the outcome measure. Unfortunately, it is often difficult to differentiate cardiac disease from systemic fluid overload as the primary reason of hospitalization. Based on our result, it seems possible that circulating bacterial DNA fragment contribute to the development of systemic fluid overload as well as atherosclerotic diseases. Nonetheless, correlations demonstrated in our observational study does not prove causation, which needs to be tested by further intervention trials.

In theory, it is also possible that pre-existing cardiovascular disease predisposes to higher levels of plasma bacterial DNA as well as future cardiovascular events. Nonetheless, we observed no association between pre-existing cardiovascular disease and plasma bacterial DNA levels. Unfortunately, we do not have baseline measurement of left ventricular function to determine its association with plasma bacterial DNA levels.

In summary, we found that circulating bacterial DNA fragment level is a strong predictor of cardiovascular event, need of hospitalization, as well as the progressive change in arterial stiffness in new PD patients. Further studies are needed to determine whether therapeutic interventions that lower circulating bacterial fragments levels could prevent cardiovascular disease in PD patients.

## Supporting Information

S1 Fig Kaplan-Meier plot of patient survival. Patients were divided to quartiles of plasma bacterial DNA. Quartile I had the lowest while quartile IV the highest plasma bacterial DNA level. Data are compared by the log rank test.
(TIF)
